# Bottom‐up effects of fungicides on tadpoles of the European common frog (*Rana temporaria*)

**DOI:** 10.1002/ece3.7332

**Published:** 2021-03-21

**Authors:** Mirco Bundschuh, Jochen P. Zubrod, Theo Wernicke, Marco Konschak, Leon Werner, Carsten A. Brühl, Patrick Baudy, Ralf Schulz

**Affiliations:** ^1^ iES Landau Institute for Environmental Sciences University of Koblenz‐Landau Landau Germany; ^2^ Department of Aquatic Sciences and Assessment Swedish University of Agricultural Sciences Uppsala Sweden; ^3^ Eusserthal Ecosystem Research Station University of Koblenz‐Landau Landau Germany; ^4^ Present address: UFZ Department of Ecological Chemistry Helmholtz Centre for Environmental Research Leipzig Germany

**Keywords:** fatty acid composition, food quality, fungicides, metamorphosis, tadpoles

## Abstract

Biodiversity is under pressure worldwide, with amphibians being particularly threatened. Stressors related to human activity, such as chemicals, are contributing to this decline. It remains, however, unclear whether chemicals exhibiting a fungicidal activity could indirectly affect tadpoles that depend on microbially conditioned leaf litter as food source. The indirect effect of fungicides (sum concentration of a fungicide mixture composed of azoxystrobin, carbendazim, cyprodinil, quinoxyfen, and tebuconazole: 100 µg/L) on tadpoles was assessed relative to leaf litter colonized by microbes in absence of fungicides (control) and a worst‐case scenario, that is leached leaf litter without microbial colonization. The quality of leaf litter as food for tadpoles of the European common frog (*Rana temporaria*) was characterized through neutral lipid fatty acid profiles and microbial sum parameters and verified by sublethal responses in tadpoles (i.e., feeding rate, feces production, growth, and fatty acid composition). Fungicides changed the nutritious quality of leaf litter likely through alterations in leaves’ neutral lipid fatty acid profiles (i.e., changes in some physiologically important highly unsaturated fatty acids reached more than 200%) in combination with a potential adsorption onto leaves during conditioning. These changes were reflected by differences in the development of tadpoles ultimately resulting in an earlier start of metamorphosis. Our data provide a first indication that fungicides potentially affect tadpole development indirectly through bottom‐up effects. This pathway is so far not addressed in fungicide environmental risk assessment and merits further attention.

## Graphical abstract

Tadpoles consuming leaf litter of different quality show changes in their energy reserves, more specifically the composition of fatty acids, and growth dynamics. Low‐quality food ultimately leads to a higher number of individuals entering metamorphosis.

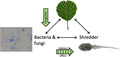

## 1 INTRODUCTION

Biodiversity is declining worldwide (Butchart et al., 2010), with amphibians being under a particular strong pressure (e.g., Scheele et al., 2019). This high pressure is underpinned by the fact that amphibians harbor more than 40% to all threatened species (IUCN, 2020). Major drivers for this global amphibian decline are invasive species, infectious diseases, the contamination, and loss of aquatic and terrestrial habitats in which amphibian realize their biphasic life cycle through land‐use changes (Collins & Storfer, 2003; Storfer, 2003). In agricultural landscapes, agrochemicals such as pesticides can represent a significant risk for local amphibian populations. Indeed, amphibians migrating in the postbreeding period through intensively used agricultural landscapes exhibit a high probability of being exposed to pesticides (Leeb et al., 2020), which can lead to significant mortality (Brühl et al., 2013). In addition, during their aquatic life stages, amphibians inhabiting ponds in the agriculturally used area (Knutson et al., 2004) may experience waterborne exposure with implications, amongst others, in their reproductive capacity (e.g., Adams et al., 2020).

Consequently, exploring the impact of pesticides on amphibians has attracted substantial scientific interest (Brühl et al., 2011). Pesticides (i.e., the fungicides pyrimethanil and tebuconazole) have, for instance, been shown to affect the metamorphosis success of the Italian tree frog (*Hyla intermedia*) exposed for more than 10 weeks to 5 and 50 µg/L (Bernabo et al., 2016). Besides the direct effects of waterborne exposure, a number of studies highlight indirect effects on tadpoles through top‐down and bottom‐up pathways. The insecticide carbaryl reduced predation on tadpoles of *Rana catesbeiana*. Although this insecticide reduced survival of tadpoles, their development and size at metamorphosis was enhanced (Boone & Semlitsch, 2003). As documented in another study, the carbaryl‐induced reduction of predation pressure can aggravate the competition for resources (i.e., periphyton). As a consequence of limited resource availability, the size of metamorphs can finally be reduced (Mills & Semlitsch, 2004). On the other hand, herbicides, which primarily interact with photoautotrophic organisms, can impact periphyton, an important food source for some tadpole species (Altig et al., 2007). This has consequences for species competing for the same resource reducing growth of tadpoles (i.e., their mass) as well as their ability to avoid predators (Rohr & Crumrine, 2005). Similarly, malathion, an insecticide, induced a complex effect cascade impacting periphyton growth, delaying the development of leopard frog tadpoles (*Rana pipiens*), and finally causing mortality when the ecosystem dried out (Relyea & Diecks, 2008). This selection of studies suggests that pesticides with clearly distinct target groups (i.e., insecticides vs. herbicides vs. fungicides) can affect amphibians directly or indirectly during the aquatic part of their life cycle.

Although the feeding strategy of tadpoles involves herbivory and detritivory (Altig et al., 2007; Whiles et al., 2010), most of the available ecotoxicological studies targeting bottom‐up effects involve the first (e.g., Rowe et al., 2001) but ignored the latter. In fact, tadpoles are recognized in their role in leaf decomposition especially in tropical streams that have generally a lower macroinvertebrate shredder diversity and abundance compared to temperate systems but a higher diversity and abundance of tadpoles (Rugenski et al., 2012). Tadpoles can also indirectly affect leaf decomposition by influencing microbial communities and macroinvertebrate feeding (Iwai et al., 2009). Moreover, a range of publications highlights the importance of leaf litter and its quality for the development of tadpoles; leaves’ phenolic and nitrogen content negatively and positively correlate with tadpole performance (e.g., Earl & Semlitsch, 2015; Iwai et al., 2009; Maerz et al., 2010; McMahon et al., 2012; Stephens et al., 2015, 2017; Stoler & Relyea, 2011). Another parameter characterizing food quality, which received relatively little attention so far, is the composition of fatty acids, particularly long‐chain polyunsaturated fatty acids. Research highlights the importance of omega‐3 fatty acids, such as eicosapentaenoic acid (EPA; 20:5ω3) and docosahexaenoic acid (DHA; 22:6ω3) as well as their precursor alpha‐linoleic acid (ALA; 18:3ω3) for animals. Those fatty acids ensure both structure and fluidity of cell membranes and support development as well as functioning of neural and ocular tissue (reviewed in Hixson et al., 2015; Twining et al., 2016). As aquatic vertebrates are not (or to a very limited share) able to synthesize EPA and DHA from ALA, their diet is (independent of their feeding guild) proposed as major source for these polyunsaturated fatty acids (Twining et al., 2016). Little is, however, known whether and to which extent chemical stressors such as fungicides can modify the nutritious quality of leaf litter for tadpoles. Fungicides change the leaf‐associated microbial community structure and as a consequence of this shift the composition of fatty acids, which might have implications on species depending on leaf litter as food sources. Comparable effects have been documented for leaf‐shredding invertebrates (Feckler et al., 2016; Konschak et al., 2019; Zubrod et al., 2011; Zubrod, Englert, Wolfram, et al., 2015).

**FIGURE 1:**
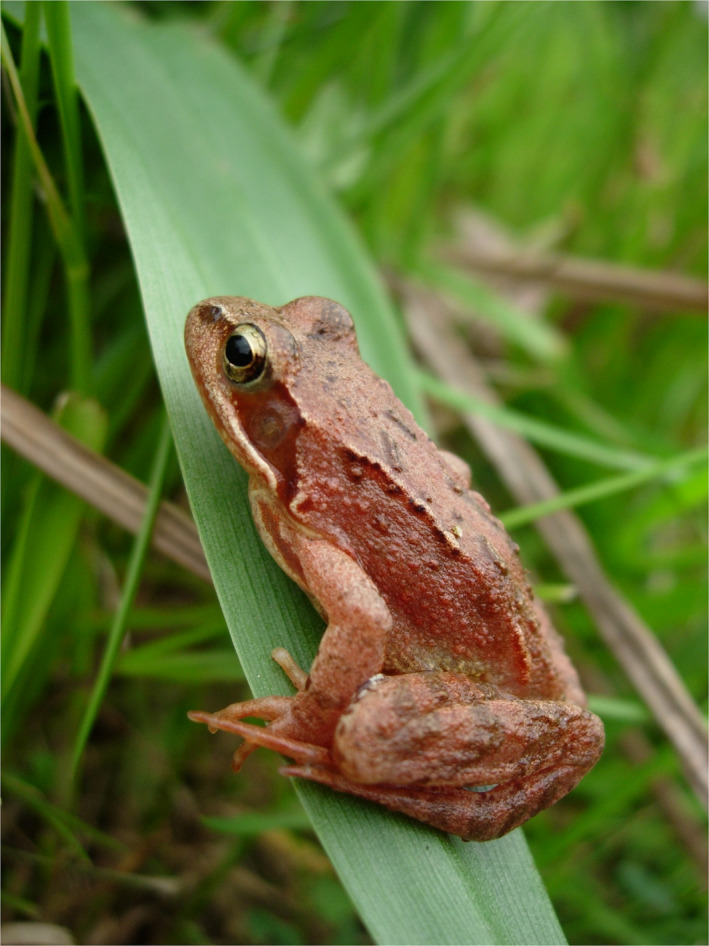
Picture of the test species *Rana temporaria* in the field. Picture by C.A. Brühl

We hypothesized that fungicide presence during microbial conditioning will change the nutritious quality of leaf litter by affecting microbial sum parameters (fungal biomass and bacterial cell counts) as well as fatty acid composition. Such modifications in food quality could ultimately translate to changes in the development of tadpoles of our test species, the European common frog (*Rana temporaria*; Figure 1). The impact induced by fungicide exposure on nutritious quality and indirectly on tadpole development was additionally compared to an assumed worst‐case scenario, namely leached leaf litter without a microbial biofilm and therefore containing no fungal biomass and bacterial cells as well as low levels of highly unsaturated fatty acids (Hixson et al., 2015).

## 2 MATERIALS AND METHODS

### 2.1 Test organisms

Two egg clutches of the European common frog (*R. temporaria*) containing roughly 130 eggs each were sampled from a pond within a protected landscape area in Southern Germany (Bienwald, 49°01′19″N, 8°10′46″E on March 21, 2016). Sampling took place with permission of local authorities (Struktur‐ und Genehmigungsdirektion Süd). In the laboratory, egg clutches were kept in pond water at 4 ± 1°C for 9 days delaying hatching and giving time for required experimental preparation. Subsequently, clutches were moved to a climate chamber set at 17 ± 1°C with a light regime as used during the experiments (light:dark rhythm of 12:12 hr) and placed in 30‐L glass aquaria filled with aerated dechlorinated and bacteria‐free tap water (filtered over Pall Kleenpak™ Water Filter, Pall GmbH). Dechlorinated and bacteria‐free tap water also served as test medium and was renewed during culturing every 2–3 days. The experiment was initiated when approximately 50% of the tadpoles reached Gosner stage 25 (Gosner, 1960), which is characterized by the disappearance of external gills and the onset of active feeding. The experiment was terminated as soon as at least one treatment exceeded a metamorphosis rate of 50%.

### 2.2 Preparation of leaf litter as food for tadpoles

The preparation of leaf litter conditioned in presence (fungicide treatment) or absence (control) of the fungicide mixture (Table 1) was performed as described in detail elsewhere (Zubrod et al., 2011) with some alterations. Briefly, leaf litter (roughly 2 kg wet weight of leaves) was sampled from the same pond as the egg clutches serving as source of a leaf‐associated microbial community characteristic for the habitats of the test species. In the laboratory, another 2 kg fresh weight of *Alnus glutinosa* (L.) GAERTN (black alder) leaves (collected from riparian trees near Landau [49°11′N, 8°05′E] during autumn 2015) were added to the inoculum and cultured in aerated nutrient medium (Dang et al., 2005) at 16 ± 1°C for three weeks. Thereby, leaves exhibiting different stages of decomposition were generated increasing microbial diversity.

**TABLE 1:** Classification, origin, ecotoxicologically relevant information, time to 50% degradation in sediment‐water systems (DT_50_), and tested nominal and measured (means with 95% confidence intervals) concentrations of the assessed fungicides

Fungicide	Chemical family	Product applied	Supplier	Mode of action^a^	Tested conc. (µg/L)	Measured conc. (µg/L)	Maximum surface water conc. (µg/L)	Chronic *Daphnia* NOEC (µg/L)^b^	DT_50_ (days)^b^
Azoxystrobin	Strobilurins	Ortiva	Syngenta Agro GmbH	Inhibition of mitochondrial respiration	20	19.5 (17.8 to 21.9)	29.7^c^	44	200
Carbendazim	Benzimidazoles	Derosal	Omya Agro GmbH	Inhibition of mitosis and cell division	20	18.4 (16.8–20.0)	1.6^d^	1.5	34
Cyprodinil	Anilino‐pyrimidines	Chorus	Syngenta Agro GmbH	Inhibition of amino acid and protein synthesis	20	18.0 (13.5–22.5)	2.2^d^	8.8	142
Quinoxyfen	Quinolines	Fortress 250	Dow AgroSciences GmbH	Perturbation of signal transduction	20	21.6 (20.8–22.0)	0.02^e^	28	127
Tebuconazole	Triazoles	Folicur	Bayer CropScience	Inhibition of sterol biosynthesis	20	22.0 (16.0–26.0)	9.11^c^	10	365

^a^ Fungicide Resistance Action Committee (2013).

^b^ FOOTPRINT (2013).

^c^ Berenzen et al. (2005).

^d^ Süss et al. (2006).

^e^ Landesamt für Umwelt, Wasserwirtschaft und Gewerbeaufsicht Rheinland‐Pfalz (2011).

This inoculum was used to condition leaf strips (4 × 7 cm^2^) cut from fresh black alder leaves either in presence (fungicide treatment) or absence (control) of a fungicide mixture (see chapter 2.3 and Table 1). Up to 150 leaf strips were conditioned in circular aquaria containing 12 L nutrient medium and 50 g wet weight of inoculum at 16 ± 1°C and in total darkness for 3 weeks under aeration. Medium with or without the fungicide mixture was entirely renewed every 7 days. For each week of the experiment, a new conditioning run (eleven in total) was started to ensure a constant provision of food of similar quality. At the start of each conditioning run, a 10‐ml water sample was collected from each aquarium and frozen for a subsequent verification of exposure concentrations (see chapter 2.4). Moreover, a subsample of leaves was conserved for characterizing the leaf‐associated microbial community by determining the bacterial cell number and fungal biomass.

Leaf litter serving as food for tadpoles in the leaching treatment was generated 4 days before its use in the feeding experiment, namely before test initiation and water exchanges, respectively. The leaching before the introduction into the test system ensured that tadpoles were not directly exposed to leachates that may contain potentially toxic phenols and secondary plant compounds (Swain, 1977). Leaching was realized in a similar setting as detailed above, while no inoculum was added and the medium was autoclaved (and thus sterilized) before use. The resulting leaf litter was not microbially conditioned but leached and served as a worst‐case scenario when it comes to food quality for shredders (e.g., Bärlocher, 1985). Leaves from the control, the leaching, and the fungicide treatment were preserved for fatty acid analyses, serving as another indicator of food quality.

### 2.3 Feeding experiment

During the experiment, three treatments were established: The treatment groups covered different food qualities, with leaf litter conditioned in absence of the fungicide mixture serving as control and those conditioned in presence of the fungicide mixture assessing indirect fungicide effects. Additionally, one worst‐case scenario was assessed exhibiting low food quality, namely leached leaf litter without a microbial biofilm. The experiment was divided in two phases: during the first phase, tadpoles were cultured individually in glass beakers, and during a subsequent second phase, they were cultured in groups of five in larger aquaria including a terrestrial area to avoid drowning of organisms after metamorphosis. Experiments were run in both phases with a light:dark rhythm of 12:12 hr at room temperature (17–21°C as documented by a temperature logger, EBI 20, Ebro). In the following, the details of the two phases are outlined.

From the approximately 200 tadpoles that successfully hatched, 99 were randomly split among the three treatment groups resulting in 33 organisms at the start of the experiment. This number should ensure the availability of sufficient individuals for neutral lipid fatty acid analyses before metamorphosis and continue the assessment of tadpole development. The first phase of the experiment was realized in 250‐ml glass beakers filled with 200 ml of test medium (see above). Each beaker was equipped with a cylindrical cage made of stainless steel mesh (mesh size: 0.5 mm) in which one tadpole was kept resulting in an independent replication of 33. For each replicate, six leaf disks (2 cm in diameter) were cut from three leaf strips of the respective treatment, with one leaf disk from each side of the main vain. Three leaf disks were offered the tadpoles as food, and the three corresponding leaf disks were stored unavailable for the tadpoles correcting for microbial and physical leaf mass losses during the feeding duration. Those leaf disks were placed in a small cuboid cage made of the same stainless steel mesh screen located below the cylindric cage in the same glass beaker (see for more details and a schematic overview Zubrod, Englert, Rosenfeldt, et al., 2015). Both cages were separated by a watch glass preventing adsorption of feces onto leaves designated to assess microbial and physical leaf mass losses. Medium was aerated throughout the study and entirely exchanged, together with the leaves on a weekly basis. At the start of the experiment and the time of water exchange, tadpoles were checked for their developmental stage and photographed. Total length (i.e., from the tip of the head to the end of the tail) was determined with MeasureMaster (mainview Software). To quantify the dry weight of leaf disk remains, they were dried for 24 hr at 60°C and weighted to the nearest 0.01 mg (Mettler Toledo, XA105). Leaf consumption was calculated as the difference between the leaf disks kept in the cuboid and cylindric cages of the same replicate. This difference was normalized to the mean length of the respective tadpole for the covered period and day. The amount of feces produced was quantified by filtering the medium at the time of water exchange through an incinerated (500°C) glass fiber filter of known dry weight. Subsequently, filters were dried (24 hr at 60°C) and weighed (nearest 0.01 mg). By computing the difference between the fresh and loaded filter, feces production was calculated. Three beakers containing medium, leaf disks but no tadpoles served to correct for the microbial production of fine particles in‐between water exchanges.

After 7 weeks, and thus the appearance of individuals at Gosner stage 41, the test set‐up was changed from an individual‐ to a group‐based setting (second phase; motivation see above). Each treatment group had four replicate polypropylene aquaria comprising 1 L of medium, a terrestrial landing area and five individuals (*n* = 4 per treatment). In other words, from the initially 33 individuals, 20 were continued in the second phase of the experiment divided in four replicates per treatment. The 13 individuals per treatment not required in the second phase were euthanized according to the German Federal Ministry of Justice and Consumer Protection, in 200 mg MS 222/L in medium adjusted to pH 7. Euthanized tadpoles were stored at −80°C until analyses of their fatty acid profile. During the second phase of this experiment, tadpoles were fed ad libitum with approximately 2 g (wet weight) leaf litter in the form of leaf strips, whose dry weight was estimated through a correction factor based on five wet‐dry‐weight measurements. In weekly intervals, the food and medium was replaced and final leaf dry mass was determined as detailed above. Similarly, tadpole length and feces production was quantified as described for the first experimental phase. The experiment was terminated after 11 weeks as no further growth of tadpoles was observed for 28 days.

### 2.4 Fungicides

The concentration of each component of the fungicide mixture (Table 1) is informed by the authors’ earlier work, suggesting implications in leaf‐associated microbial communities at the concentration applied here (Zubrod, Englert, Wolfram, et al., 2015). Moreover, the levels of most fungicides are close to field measured concentrations or regulatory relevant effect thresholds, such as the no observed effect concentration in the standard test species *Daphnia magna* (Table 1). Water samples were taken during the microbial conditioning, preserved at −20°C and chemically analyzed (i.e., quantification of fungicide concentrations) using ultra‐high‐performance liquid chromatography‐mass spectrometry (Thermo Fisher Scientific). Since measured initial concentrations deviated by a maximum of 20% from the nominal ones (Table 1), the paper is based on the latter.

### 2.5 Microbial parameters

Leaf‐associated microbial communities were characterized using fungal biomass and bacterial cell numbers as descriptors. Fungal biomass was determined using ergosterol as proxy following Gessner (2005). Briefly, ergosterol was extracted from freeze‐dried leaf material of known dry weight in alkaline methanol at 80°C. Following purification by solid phase extraction (Sep‐Pak^®^ Vac RC tC18, 500 mg, Waters GmbH), ergosterol was quantified by high‐performance liquid chromatography (1,200 Series, Agilent Technologies).

To quantify the number of leaf‐associated bacterial cells, three formalin preserved leaf disks were treated with ultrasound to dislodge bacteria (see for details Buesing, 2005). Cells were subsequently stained with SYBRGreen II (Molecular Probes) and photographed under an epifluorescence microscope (Axio Scope.A1, AxioCam MRm). Using an image analysis software (AxioVision, Carl Zeiss MicroImaging), cells were counted. Both ergosterol and bacterial cell counts were normalized to leaf dry mass before statistical analyses.

### 2.6 Fatty acid analysis

Fatty acids were analyzed as detailed by Konschak et al. (2020). The focus in tadpoles was on triacylglycerol fatty acids (i.e., neutral lipid fatty acids, NLFAs) constituting the major energy storage (Arts et al., 2009) whose composition is affected relatively quickly (compared to phospholipid fatty acids) by changes in the diet (Iverson, 2012). As fatty acids serve as proxy determining the basal microbial community structure for conditioned leaves as well as for the microbial activity (Torres‐Ruiz & Wehr, 2010, 2020), NLFAs associated with leaves were analyzed, too. NLFAs from leaf material and tadpoles were extracted after freeze‐drying with a chloroform/methanol/Milli‐Q‐water mixture (1:2:0.8, V/V/V). NLFAs were separated and purified using solid phase extraction (Chromabond^®^ Easy polypropylene columns, Macherey‐Nagel). Samples were concentrated under a nitrogen atmosphere at 40°C using a dry bath heater (EC‐1V‐130, VLM). Transesterification of NLFAs to fatty acid methyl esters was conducted with trimethylsulfonium hydroxide (TMSH, 0.25 M in methanol, for gas chromatography derivatization, Sigma‐Aldrich).

Samples were measured by gas chromatography with a flame ionization detector (GC‐FID, Varian CP‐3800, Agilent) equipped with a capillary column (J&W DB‐225, length: ~30 m, diameter: 0.25 mm, film thickness: 0.25 µm, Agilent) using nitrogen as carrier gas. NLFAs were identified via the retention time of the respective methyl ester derivate by using an external standard containing 38 FAMEs with known concentrations (Sigma‐Aldrich). NLFA concentrations were quantified by using a seven‐point external standard curve (Supelco 37 Component FAME Mix, Sigma‐Aldrich) and adjusted via blank correction and recovery by adding a deuterium‐marked internal standard (Tristearin‐D105, Larodan) prior to the extraction from leaves and tadpoles. The resulting concentrations were extrapolated to the samples’ volume.

### 2.7 Calculations and statistics

Food consumption and feces production were normalized for tadpole length and day by following Zubrod et al. (2011). Ergosterol concentrations, bacterial cell numbers, and individual NLFAs were normalized for sample dry weight. To test for statistical differences among treatments, analyses of variance (ANOVA) followed by Dunnett's test as post hoc test or *t* test were performed, respectively. Concentrations of individual NLFAs (µg/mg) were square‐root transformed to reduce the weight of highly abundant fatty acids. To test for significant differences among NLFA profiles, permutative multivariate analysis of variance (PERMANOVA) was performed. Prior to testing for differences in NLFA profiles, data were checked for homogeneity of variances in order to exclude statistically significant differences stemming from within‐group variability (i.e., dispersion effect). For PERMANOVA, 999 permutations and a Bray–Curtis dissimilarity matrix were used. To test for statistically significant differences between each treatment and the control of each individual NLFA, normality and variance homogeneity were tested first employing Shapiro–Wilks and Levene's test, respectively. Subsequently, one‐way ANOVAs followed by Dunnett's test or Wilcoxon rank‐sum test with Bonferroni adjustment as their nonparametric alternative were employed. NLFA composition in leaves and tadpoles was visualized using nonmetric multidimensional scaling (NMDS) calculated with a Bray–Curtis dissimilarity matrix, and a stress function ranging from 0 to 1 was used as a goodness‐of‐fit measure. To estimate time to metamorphosis, we used the Kaplan–Meier estimator, a nonparametric method to estimate survival functions (i.e., probability of individuals to go into metamorphosis). As individuals could be followed throughout the entire experiment (i.e., both phases) and the random effect introduced by the second phase was deemed rather unimportant (e.g., ad libitum feeding, no increase in density), we opted for this approach instead of more complex modeling. Both the leaching and the fungicide treatment were finally compared to the control using the Peto & Peto modification of the Gehan‐Wilcoxon test. All calculations, statistics, and data visualizations were performed using the statistics software R (3.3.2) with the add‐on packages “multcomp“ (1.4‐16), “vegan” (2.5‐5), “car” (3.0‐3), “ggpubr” (0.2), and “ggplot2” (3.1.1). The significance level was set at α < 0.05. To cope with criticism of null hypothesis significance testing, data interpretation is based on both statistical significance and effect sizes (Newman, 2008).

## 3 RESULTS AND DISCUSSION

### 3.1 Tadpole food quality

**FIGURE 2:**
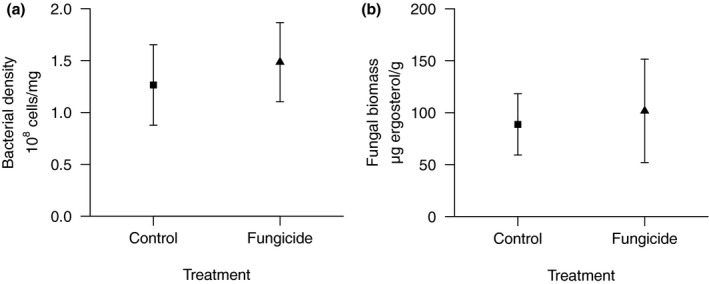
Leaf‐associated microbial community characterized by means (with 95% confidence intervals) of bacterial cell density (a) and fungal biomass (b). Differences are not statistically significant. Leaves from the leaching treatment did not show any relevant values and are thus not represented

Microbial parameters (fungal biomass and bacterial abundance; Figure 2) and NLFA profiles as proxies for the basal microbial community structure deviated (*p* >> .05 in PERMANOVA with Bonferroni adjustment, Table 2; Figure S1) only slightly between the leaf litter conditioned in absence (control) and leaf litter conditioned in presence of the fungicide mixture. In fact, neither the sum concentration of all NLFAs nor any individual NLFA deviated statistically significantly between these two food sources. Nonetheless, the effect sizes for some individual NLFAs exceeded 30% (Table 2). Although highly unsaturated fatty acids contributed, according to SIMPER analysis, only approximately 7% to the differences between these food types (Table 2), EPA (20:5ω3, difference of 36%) and DHA (20:6ω3, difference of 226%) were substantially elevated (Table 2) suggesting higher quality of fungicide‐treated leaf litter (see also Feckler et al., 2016; Zubrod et al., 2017).

**TABLE 2:** Concentration of individual neutral lipid fatty acids (NLFAs) in control, leached, or fungicide‐treated leaves

	Leaves							Tadpoles						
	Control	Leaching	Explained deviation from the control (%) according to SIMPER	Difference (%) relative to the control	Fungicide	Explained deviation from the control (%) according to SIMPER	Difference (%) relative to the control	Control	Leaching	Explained deviation from the control (%) according to SIMPER	Difference (%) relative to the control	Fungicide	Explained deviation from the control (%) according to SIMPER	Difference (%) relative to the control
*Concentration of fatty acids in µg/mg*														
12:0	0.07	0.12	0.28	+61	0.06	0.57	−23	0.27	0.45	1.23	+67	0.17	1.67	−37
13:0	0.01	0.01	0.03	−51	0.01	0.07	+28	ND	ND	—		ND	—	
14:0	0.41	0.30	0.49	−27	0.41	1.86	−2	0.43	0.71	1.51	+67	0.28	1.62	−35
15:0	0.07	0.04	0.11	−38	0.07	0.40	−2	0.21	0.46*	0.91	+116	0.13	0.79	−38
16:0	3.00	6.00*	12.39	+100	2.40	14.63	−20	6.30	9.96	16.96	+58	3.94*	17.73	−38
16:1ω7	0.63	0.15*	2.00	−77	0.50	3.13	−20	0.86	1.67	3.01	+93	0.53	3.06	−39
17:0	0.10	0.34*	0.99	+240	0.08	0.63	−23	0.28	0.50	0.84	+78	0.20	0.73	−29
18:0	0.61	0.71	0.42	+16	0.49	1.93	−19	3.72	5.23	9.52	+41	2.64	10.15	−29
18:1ω7	0.44	0.26	0.74	−41	0.40	1.70	−10	1.57	2.17	3.61	+38	1.06	4.82	−33
18:1ω9	0.62	0.83	1.36	+34	0.45	3.84	−27	2.17	2.78	4.24	+28	1.37	7.11	−37
18:2ω6	1.48	3.28*	7.25	+120	1.30	8.47	−13	4.30	6.51	13.51	+51	2.75	16.34	−36
18:3ω3	5.03	20.81*	64.32	+313	3.88	35.02	−23	2.02	9.86*	23.61	+389	1.25	9.34	−38
18:3ω6	0.02	0.01	0.05	−65	0.03	0.13	+30	0.07	0.07	0.20	+7	0.04	0.34	−48
20:0	0.88	1.43	2.60	+62	0.61	4.75	−31	0.31	0.66	1.43	+116	0.19	1.11	−37
20:1ω9	0.04	0.02	0.07	−36	0.02	0.33	−29	0.04	0.03	0.15	−10	0.00	0.31	−92
20:2ω6	0.02	0.02	0.02	−4	0.01	0.09	−35	0.06	0.07	0.18	+11	0.03*	0.34	−59
20:3ω3	0.01	0.02	0.06	+67	0.02	0.17	+10	0.16	0.47	1.05	+188	0.06	0.88	−61
20:3ω6	0.03	0.03	0.10	+10	0.03	0.19	+4	0.29	0.32	0.52	+11	0.15*	1.14	−49
20:4ω6	0.08	0.00	0.33	−100	0.08	0.66	+2	2.52	2.12	4.97	−16	1.59	8.29	−37
20:5ω3	0.06	0.01	0.24	−91	0.09	0.65	+34	1.16	1.64	2.90	+42	0.75	3.74	−35
21:0	ND	ND	—	—	ND	—	—	0.30	0.58	2.15	+90	0.05	2.16	−85
22:0	1.29	1.70	3.24	+32	0.81	6.91	−27	0.26	0.45	0.89	+72	0.20	0.87	−25
22:2ω6	0.44	0.24	0.92	−46	0.33	3.62	−25	0.06	0.10	0.26	+82	0.05	0.28	−7
22:6ω3	0.15	0.02	0.56	−85	0.51	6.43	+226	2.47	1.94	5.83	−22	2.38	7.31	−4
23:0	0.13	0.09	0.19	−23	0.14	1.01	+7	ND	ND	—		ND	—	
24:0	0.44	0.50	1.24	+13	0.29	2.80	−36	0.17	0.20	0.52	+20	0.09	0.87	−48
Total NLFAs	16.07	36.91*	—	+130	13.00	—	−19	30.00	48.95	—	+63	19.89*	—	−34

Note

The concentration of NLFAs in tadpoles sampled after 49 days being fed with one of these leaf litter types is also reported. Additionally, the contribution to the dissimilarity (according to SIMPER analyses) of NLFA profiles in the leaching and fungicide treatment relative to the control is displayed. Asterisks indicate a statistically significant difference to the control (*p* < .05).

Statistically significant differences were observed between control and leached *Alnus* leaves (Table 2; Figure S1): The total concentration of NLFAs was twice as high in leached leaves, with these differences being mainly (>70% according to SIMPER) triggered by higher levels of the precursors for ARA, EPA, and DHA, namely LAN (18:2ω6) and ALA (18:3ω3). As ALA is considered a marker of photoautotrophic and terrestrial origin (Hixson et al., 2015), high levels are expected in leaf material. Despite the ability of leaf‐associated fungi to synthesize these precursors (Arce Funck et al., 2015), a rapid decline in both LAN and ALA, as observed for control leaves, is a consequence of microbial colonization (Torres‐Ruiz & Wehr, 2010). At the same time, the essential omega three and six fatty acids ARA (20:4ω6), EPA (20:5ω3), and DHA (22:6ω3) were substantially lower in leached leaf litter. Driven by their low contribution to NLFA levels in general, these deviations contributed only little more than 1% to the overall differences in NLFA profiles (Table 2).

Nonetheless, these data suggest that heterotrophic microorganisms are capable of increasing the concentrations of these highly unsaturated fatty acids by a factor of up to 25, a pattern which was less evident in literature (Torres‐Ruiz & Wehr, 2010, 2020). These insights contradict earlier studies highlighting that ARA, EPA, and DHA could not be detected in monocultures of leaf‐associated fungi grown in nutrient medium (Arce Funck et al., 2015). The substantially higher levels of these highly unsaturated fatty acids on leaves after microbial conditioning (both control and fungicide treatment) may be explained by the unnoticed presence of diatoms or microinvertebrates for which these fatty acids are considered indicative (Torres‐Ruiz & Wehr, 2020). Since the availability of such fatty acids in the diet of invertebrates and vertebrates is, as detailed in the introduction, central for their development (reviewed for vertebrates in general by Hixson et al., 2015; Twining et al., 2016), it is of paramount importance to understand the underlying processes and how chemicals could interfere with those.

### 3.2 Reponses of tadpoles

**FIGURE 3:**
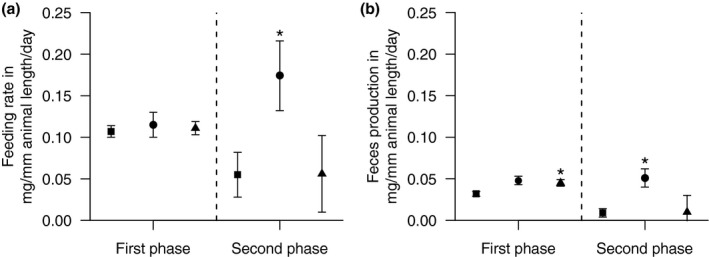
(a) Mean feeding rate and feces production (both with 95% confidence intervals) of tadpoles fed with control (squares), leached (circles), or fungicide‐treated (triangle) leaves during the first and the second phase of the experiment. Asterisks denote statistically significant differences relative to the control

Irrespective of the three offered food qualities (see above), tadpoles consumed similar amounts of leaf material during the first experimental phase (Figure 3a). Despite the statistically significantly higher feces production in treatments receiving leached leaves and those from the fungicide treatment (Figure 3b), assimilation (here defined as the difference between consumption and feces production) deviated by only around 5% suggesting minor differences in nutrient uptake. On the contrary, assimilation was higher only for organisms feeding on leached leaf litter during the second experimental phase, which was driven by a higher leaf consumption (Figure 3a) that in turn may be triggered by inter‐specific facilitation (Jonsson & Malmqvist, 2003). These observations suggest that tadpoles of the European common frog are not in all phases of their aquatic life cycle capable of compensating for low food quality through changes in their digestive physiology or increased consumption (Raubenheimer & Simpson, 1998) implicating impacts in their development.

**FIGURE 4:**
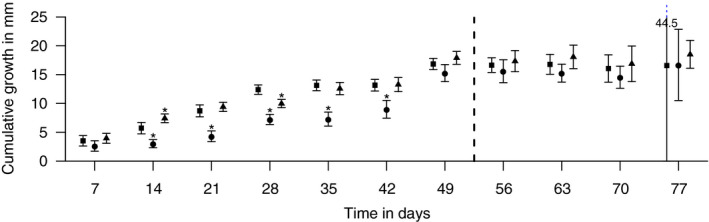
Mean growth (with 95% confidence intervals) of tadpoles in intervals of 7 days relative to the start of the experiment. Tadpoles were fed with control (squares), leached (circles), or fungicide‐treated (triangle) leaves during the first and the second phase of the experiment. The dashed line indicates the switch from the first to the second phase of the experiment. Asterisks denote statistically significant differences relative to the control

Indeed, tadpoles showed the quickest growth when fed with microbially conditioned leaves—irrespective whether treated with fungicides or not—(Figure 4) supporting the assumption that microbially conditioned leaf litter allows tadpoles to develop (Stone & Mohammed, 2017). Those organisms fed with fungicide‐treated leaves grew bigger after 14 days, while this observation was reversed after 28 days (Figure 4). These slight but statistically significant differences in growth relative to those organisms fed with control leaves might be triggered by differences in microbial communities and ultimately NLFA profile (Table 2). As differences in those variables are, as detailed above, statistically not significant, another explanation is the adsorption of fungicides on the leaf litter during conditioning. In combination with the stability of these fungicides to degradation (Table 1), this points to an ultimate exposure and potential effect on tadpoles through dietary uptake, which is also hypothesized for invertebrate shredders as an alternative effect pathway (Zubrod, Englert, Wolfram, et al., 2015). Irrespective of the underlying pathway, changes in food quality triggered a 30% reduction of the total NLFA concentrations in tadpoles fed with fungicide‐treated leaves relative to the control. This observation suggests either a lower energy assimilation due to a lower food quality or, according to the dynamic energy budget theory (Kooijman, 2010), higher energetic expenditures for detoxification and damage repair induced by the fungicide stress. If true, such a higher cost in maintenance confirms an exposure to fungicides indirectly. Tadpoles fed with leached leaves without biofilm, in contrast, grew substantially slower relative to the other treatments until day 42 (Figure 4).

**FIGURE 5:**
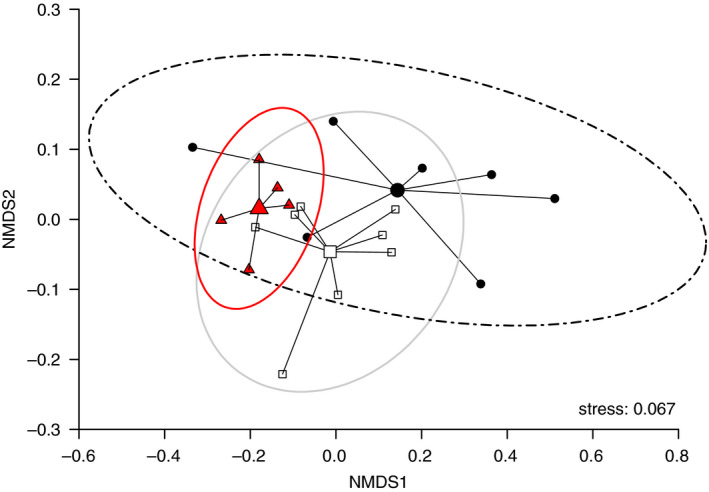
Nonmetric multidimensional scaling (NMDS) ordination (with 95% confidence ellipses) for the composition of all detected NLFAs in the tadpoles measured after 49 days (*n* = 5–7) control (open squares), leached (black circles) or fungicide‐treated (red triangles) leaves. The stress value is displayed as a “goodness‐of‐fit” measure of the NDMS, with values below 0.2 indicating a reasonable fit

The differences in growth during the first experimental phase are also partly reflected by statistically significant changes in the NLFA profile of tadpoles among treatments at the time individuals at Gosner stage 41 appeared (*p* = .019 in PERMANOVA; Figure 5). Although the statistically significant outcome might be the result of a dispersion effect (see overlapping 95%‐confidence ellipses in Figure 5), the concentration of individual NFLAs deviated between treatments. The shift in the leaching treatment is largely explained by a five‐fold higher concentration of 18:3ω3 (ALA) fatty acid (Table 2) contributing, according to SIMPER analyses, around 25% to the differences in the FA profile relative to the other two treatments. The retarded growth in the leaching treatment seems mainly related to food of low quality (Table 2), more specifically the lack of dietary provisioning of highly unsaturated fatty acids such as ARA, EPA, and DHA. Since tadpoles sampled after 49 days from all treatments show, however, no difference in the concentration of these three fatty acids, despite partly substantial deviations in dietary concentrations, it may be assumed that tadpoles are capable of converting ALA to EPA and DHA as well as LIN to ARA—a process that is suggested to be rare in organisms (Fritz et al., 2019), inefficient and thus energetically costly (Twining et al., 2016).

**FIGURE 6:**
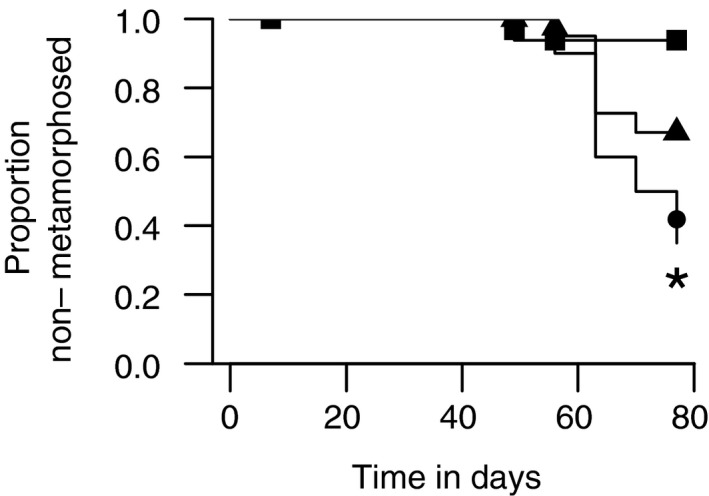
Proportion of organisms remaining in the larval stage over the study duration of 11 weeks. Tadpoles were fed with control (squares), leached (circles), or fungicide‐treated (triangle) leaves. The asterisk denotes a statistically significant difference relative to the control

During the second experimental phase, tadpole growth stagnated (independent of the treatment) suggesting that the impact of food quality was limited to growth but allowed the test species to still reach the size critical for metamorphosis. The long lack phase, however, indicates that leaf litter as a sole food source may be insufficient to optimally support tadpole metamorphosis warranting further research to address the nutritious requirements in this sensitive phase of the amphibian life cycle. The rate of metamorphosing tadpoles, for instance, was lowest for the control and with around 50% highest for organisms that fed on leached leaf litter (Figure 6). The observations for fungicide‐treated (about 30% metamorphosis) and leached leaves could point to unfavorable conditions in the aquatic environment triggering an early metamorphosis (Figueiredo & Rodrigues, 2014; Nataraj & Krishnamurthy, 2012) aiming for more favorable conditions in the terrestrial system.

## 4 CONCLUSION

The present study shows that fungicides can indeed induce some alterations in the quality of leaf litter serving as food for tadpoles by changing the NLFA profile (indirect effect) and by potentially adsorbing onto the ingested material (direct effect), though not always statistically significant. The ultimate implications of fungicides on tadpole growth have been limited but led to a higher metamorphosis rate (30%) relative to the control (5%) at the termination of the experiment (11 weeks). Such changes in the metamorphosis pattern might, however, decouple the subsidy of terrestrial food webs by aquatic resources due to a temporal mismatch of requirements and availability (Schulz et al., 2015). Consequently, our study indicates that fungicides can affect tadpoles and that antimicrobials in general could be contributing to a lower fitness of amphibian populations and hence their resistance to drivers of biodiversity loss. However, the differences uncovered in the present study are often statistically nonsignificant and partly subtle in nature, which calls for a more systematic assessment of the relevance of the exemplified effect pathway.

## CONFLICT OF INTEREST

Several authors are fully or partially involved in the private sectors. We however see no conflict of interest arising from this involvement for the present submission.

## AUTHOR CONTRIBUTION

**Mirco Bundschuh:** Conceptualization (equal); Formal analysis (equal); Funding acquisition (supporting); Supervision (equal); Writing‐original draft (lead); Writing‐review & editing (equal). **Jochen Zubrod:** Conceptualization (equal); Formal analysis (equal); Supervision (equal); Visualization (lead); Writing‐review & editing (equal). **Theo Wernicke:** Data curation (lead); Writing‐review & editing (equal). **Marco Konschak:** Data curation (supporting); Formal analysis (supporting); Investigation (supporting); Visualization (supporting); Writing‐review & editing (equal). **Leon Werner:** Investigation (supporting); Writing‐review & editing (supporting). **Carsten A. Brühl:** Funding acquisition (supporting); Writing‐review & editing (equal). **Patrick Baudy:** Data curation (equal); Investigation (supporting); Writing‐review & editing (equal). **Ralf Schulz:** Conceptualization (equal); Funding acquisition (lead); Writing‐review & editing (equal).

## ETHICAL APPROVAL

The experiment was authorized by the Federal Investigation Office (Landesuntersuchungsamt) according to § 8a of the German law for animal welfare with license number 23 177‐07/G 18‐20‐009 (NTP‐ID: 00021023‐1‐5), the Institutional Animal Care and Use Committee at the University of Koblenz‐Landau and the Struktur‐ und Genehmigungsdirektion Süd (Neustadt an der Weinstraße, Germany) for extraction of animals from the wild.

## Supporting information

Fig S1

## Data Availability

Data is available from dryad entitled “Data related to: Bottom‐up effects of fungicides on tadpoles of the European common frog (Rana temporaria)”: https://doi.org/10.5061/dryad.2rbnzs7mh.
