# Supplementary material for: Bottom‐up effects of fungicides on tadpoles of the European common frog (Rana temporaria)
Source: Ecol Evol. 2021 Mar 21;11(9):4353–65. doi: 10.1002/ece3.7332 (PMC8093721; doi:10.1002/ece3.7332)
Supplement: Supplementary file 1 — Fig S1 [file ECE3-11-4353-s001.docx]

**Supporting Information**

**Bottom-up effects of fungicides on tadpoles of the European common frog (*Rana temporaria*)**

Mirco Bundschuh^1,2,*^, Jochen P. Zubrod^1,3^, Theo Wernicke^1,a^, Marco Konschak^1^, Leon Werner^1^, Carsten A. Brühl^1^, Patrick Baudy^1^, Ralf Schulz^1,3^

^1^ iES Landau, Institute for Environmental Sciences, University of Koblenz-Landau

^2^ Department of Aquatic Sciences and Assessment, Swedish University of Agricultural Sciences

^3^ Eusserthal Ecosystem Research Station, University of Koblenz-Landau

Correspondence to:

Mirco Bundschuh

iES Landau, Institute for Environmental Sciences, University of Koblenz-Landau

Fortstraße 7

76829 Landau, Germany

[bundschuh@uni-landau.de](mailto:bundschuh@uni-landau.de)

Present address

^a^ UFZ Department of Ecological Chemistry, Helmholtz Centre for Environmental Research, Leipzig

Figure S1: Non-metric multidimensional scaling (NMDS) ordination for the composition of all detected NLFAs associated with the leaf material (n = 2-5) conditioned under control conditions (open squares), leached (black circles) or conditioned in presence of the fungicides (red triangles). The stress value is displayed as a “goodness-of- fit” measure of the NDMS, with values below 0.2 indicating a reasonable fit.
